# Electroacupuncture to treat painful diabetic neuropathy: study protocol for a three-armed, randomized, controlled pilot trial

**DOI:** 10.1186/1745-6215-14-225

**Published:** 2013-07-18

**Authors:** Seunghoon Lee, Joo-Hee Kim, Kyung-Min Shin, Jung-Eun Kim, Tae-Hun Kim, Kyung-Won Kang, Minhee Lee, So-Young Jung, Mi-Suk Shin, Ae-Ran Kim, Hyo-Ju Park, Kwon-Eui Hong, Sun-Mi Choi

**Affiliations:** 1 Acupuncture, Moxibustion & Meridian Research Group, Korea Institute of Oriental Medicine, Daejeon, Korea; 2 Department of Acupuncture & Moxibustion, College of Korean Medicine, Kyung Hee University, Seoul, Korea; 3 Mokhuri Neck and Back Hospital, Seoul, Korea; 4 Department of Acupuncture & Moxibustion, College of Korean Medicine, Daejeon University, Daejeon, Korea

**Keywords:** Electroacupuncture, Painful diabetic neuropathy, Protocol

## Abstract

**Background:**

The purpose of this study is to conduct a basic analysis of the effectiveness and safety of electroacupuncture in the treatment of painful diabetic neuropathy (PDN) as compared to placebo and usual care and to evaluate the feasibility of large-scale clinical research.

**Methods/design:**

This study is a protocol for a three-armed, randomized, patient-assessor-blinded (to the type of treatment), controlled pilot trial. Forty-five participants with a ≥ six month history of PDN and a mean weekly pain score of ≥ 4 on the 11-point Pain Intensity Numerical Rating Scale (PI-NRS) will be assigned to the electroacupuncture group (n = 15), sham group (n = 15) or usual care group (n = 15). The participants assigned to the electroacupuncture group will receive electroacupuncture (remaining for 30 minutes with a mixed current of 2 Hz/120 Hz and 80% of the bearable intensity) at 12 standard acupuncture points (bilateral ST36, GB39, SP9, SP6, LR3 and GB41) twice per week for eight weeks (a total of 16 sessions) as well as the usual care. The participants in the sham group will receive sham electroacupuncture (no electrical current will be passed to the needle, but the light will be seen, and the sound of the pulse generator will be heard by the participants) at non-acupuncture points as well as the usual care. The participants in the usual care group will not receive electroacupuncture treatment during the study period and will receive only the usual care. The follow-up will be in the 5th, 9th and 17th weeks after random allocation. The PI-NRS score assessed at the ninth week will be the primary outcome measurement used in this study. The Short-Form McGill Pain Questionnaire (SF-MPQ), a sleep disturbance score (11-point Likert scale), the Short-Form 36v2 Health Survey (SF-36), the Beck Depression Inventory (BDI) and the Patient Global Impression of Change (PGIC) will be used as outcome variables to evaluate the effectiveness of the acupuncture. Safety will be assessed at every visit.

**Discussion:**

The result of this trial will provide a basis for the effectiveness and safety of electroacupuncture for PDN.

**Trial registration:**

Clinical Research information Service. Unique identifier: KCT0000466.

## Background

Diabetic peripheral neuropathy (DPN) is the most common complication of diabetes mellitus (DM), affecting up to 50% of patients with types 1 and 2 DM [1,2]. It is reported that approximately 16% of patients with DM suffer from painful diabetic neuropathy (PDN) [3-5], and its prevalence increases as life expectancies increase [6]. The symptoms of PDN are predominantly paresthesia (sensation of numbness, prickling, tingling or burning), dysesthesia (unpleasant or abnormal sense of touch) and hyperesthesia (abnormal, increased sensitivity to normal stimuli) [7]. It is often accompanied by depression, anxiety and sleep disturbance, adversely affecting the capacity for work, health-related quality of life (HRQoL), morbidity and society costs [3].

The etiology of diabetic neuropathy is poorly understood. However, downstream metabolic cascades induced by lasting hyperglycemia have been proposed to cause peripheral nerve injury. Underlying mechanisms include an increased flux through the polyol pathway, glycation and advanced glycation end-products, oxidative stress, activation of protein kinase C and pro-inflammatory processes [8]. It is known that damage to small unmyelinated C-fibers is initiated in the early stages of diabetes [9] and that hyperactivity of these fibers results in diabetic neuropathic pain [10]. Therefore, early detection of this impairment is suggested to prevent exacerbation of neurological complications and reduce severe pain symptoms [11].

For this reason, intensive glycemic control care from the early stages of DM, including insulin treatment as initial pharmacotherapy [12] and lifestyle interventions [13], have been recommended to prevent microvascular complications. Moreover, intensive glycemic control has been shown to delay the onset or slow progression of the early stages of PDN in types 1 and 2 DM [14,15], and maintenance hemoglobin A1c (HbA1c) levels of approximately > 6.5 to 7% are recommended in light of the patient’s characteristics (for example, ethnicity, race or disease duration) [16,17]. But normal maintenance of the glycemic level is a difficult goal, and if symptoms of PDN have occurred, it is difficult to improve them through glycemic control. There are two agents for symptomatic therapy, pregabalin (antiepileptics) and duloxetine (selective serotonin/noradrenaline reuptake inhibitor, SNRI), that are approved by the Food and Drug Administration (FDA) in the USA for the treatment of PDN. Tricyclic antidepressants, antidepressants, opioids and topical agents have also been used to treat the painful symptoms of PDN [3,18]. Although some types of pain medications have been known to be effective for pain reduction, many patients with PDN are unable to achieve a level of pain relief that is greater than 30% to 50% [18], and the long-term use of these conventional medications may lead to significant side effects [19,20].

Acupuncture has long been used in East Asia for pain relief based on the Qi theory, and acupuncture can reduce pain by regulating the imbalance of Qi. In the West, acupuncture has increasingly been a recent subject of research in the treatment of chronic pain. Acupuncture analgesia might be explained by the following scientific mechanisms: local effect, mediated by adenosine A1 receptors [21]; segmental analgesia, based on the pain gate control theory [22]; extra-segmental analgesia, the releasing of opioid peptides or descending inhibitory pain control [22,23] and central regulation of the limbic system, which is relevant to the affective component of pain [22]. In a recent, well-designed meta-analysis of randomized clinical trials (RCTs) with data from 17,922 patients, it was reported that acupuncture is more effective for treating chronic pain than is sham acupuncture and no treatment [24]. With more extensive basic and clinical evidence, acupuncture is being more widely used for various types of pain control, including the pain of neuropathy.

According to a recent review [25] assessing the quality of the RCTs of acupuncture in DPN, 75 RCTs that used acupuncture as an intervention were identified for analysis through a systematic search. However, the quality of almost all of the studies was low because the researchers did not follow standard reporting guidelines, such as the Consolidated Standards for Reporting of Trials statement 2010 (CONSORT2010) and Standards for Reporting Interventions Controlled Trials of Acupuncture 2010 (STRICTA2010). No study described the mechanism of allocation concealment, applied the methods of blinding or presented detailed follow-up information, such as the study dropout rate.

Although using acupuncture for PDN is becoming more common in clinical practice, there is no evidence for the effectiveness and safety of the treatment that is supported by well-designed RCTs [3]. Our study will conduct a clinical trial with a low risk of bias to assess the effectiveness and safety of electroacupuncture for treating PDN by comparing it to a sham procedure and the usual care.

## Methods/Design

### Objective

The aims of this study are (1) to assess the effectiveness and safety of electroacupuncture in treating PDN for pain, sleep disturbance, depression, HRQoL and adverse events as compared to the usual care, (2) to evaluate the specific effect of electroacupuncture for treating PDN as compared to sham electroacupuncture, (3) to evaluate the non-specific effect of electroacupuncture for treating PDN by comparing it to sham electroacupuncture and the usual care and (4) to identify the feasibility of a large-scale clinical trial.

### Design and setting

This study is a stratified (whether or not a subject is taking pain control medication for PDN, with equal randomization), patient-assessor-blinded (to the type of treatment), controlled, three-armed parallel-group pilot study conducted in Korea.

#### Recruitment period

The participants will be recruited from the Daejeon University Hospital in Daejeon, which has a population of approximately 1,525,000. Recruitment is expected to be completed from June 2012 to July 2013.

#### Methods of recruitment

A total of 45 participants with PDN will be recruited from the outpatients of the acupuncture and moxibustion clinics of Daejeon University Hospital. We will recruit the participants by advertising in the hospital, media (local newspapers and public newsletters) and the Internet homepages of hospitals and public institutions. Public lectures on DM and PDN will be conducted in social work centers to provide information on the importance of treating PDN. Because PDN patients are rarely aware of their disease despite taking glucose control medication, we will provide information about the trial to the doctors in the public health centers and primary care facilities in which patients are treated for glucose control.

#### Study plan

**Figure 1:**
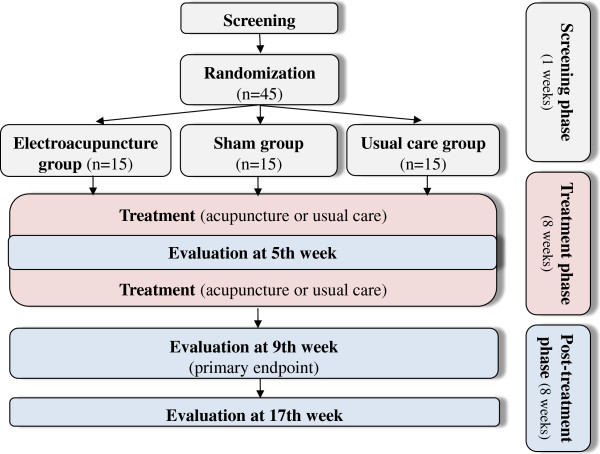
A flowchart of the study process.

Subject information will be collected after oral and written consent is obtained from each participant at the first visit. This study will be conducted in three phases (Figure 1):

1) Screening phase (one week): after a participant consents voluntarily to the study, they will be screened using the inclusion/exclusion criteria at the first visit. The participants will be asked to record a daily score in a pain diary for one week. At the end of the screening phase, if an eligible participant meets the study criteria with poorly controlled neuropathic pain (moderate to severe, consistent with stages 2 and 3 of polyneuropathy [26]), then the subject will be randomly allocated into one of three groups (electroacupuncture, sham or usual care group) with a 1:1:1 allocation ratio, and will receive eight weeks of treatment with eight weeks of follow-up.

2) Treatment phase (eight weeks): the electroacupuncture group will receive electroacupuncture treatment plus the usual care, the sham group will receive sham electroacupuncture (minimal acupuncture stimulation with no current at non-acupuncture points) plus the usual care and the usual care group will receive the usual care (an educational program and conventional medication). Two treatment sessions per week will be performed during an eight-week period for 16 sessions, and if the participants do not receive two treatments per a week, then it is possible to receive treatment within two days, but eight sessions must be completed within four weeks. During the treatment phase, the participants in all groups will be permitted to take antihyperglycemic medications (oral hypoglycemic agents or insulin injection) with no changes in the type of medication and minimal changes in the maintenance dose (≤ 25%). If the participants are taking pain control medication for PDN, that will be permitted with no change in the maintenance dose.

3) Post-treatment phase (eight weeks): the participants will be assessed after eight weeks of randomization as a primary endpoint, and the follow-up assessment will be conducted eight weeks after the primary endpoint. During the post-treatment phase, pain control medication will be allowed without limitation, but participants will be educated to record the usage of medication, and the records will be checked at the follow-up assessment. To motivate the participants to complete the study, we will offer manual acupuncture for PDN to the usual care group if they want to receive acupuncture treatment after the trial is completed.

### Types of participants

#### Inclusion criteria

Participants who meet the following conditions will be included:

1. Males and females aged 18 to 75 years;

2. A diagnosis of type 1 or 2 DM;

3. Distal symmetric lower limb pain present for at least six months;

4. A score ≥ 4 on the 11-point Pain Intensity Numerical Rating Scale (PI-NRS) for the pain of diabetic peripheral neuropathy at least four days a week before the randomization;

5. A minimum of three scores on the history and physical examination portion of the Korean version of the Michigan Neuropathy Screening Instrument (MNSI) [27];

6. A minimum of two abnormalities on the following measures:

a. vibration perception by a 128 Hz tuning fork;

a. 10 g monofilament test;

a. ankle reflexes;

7. Stable use (variation of a major drug ≤ 25%) of pain control medications for PDN in the three months prior to screening or no use of pain control medications for PDN within the past one month.

#### Exclusion criteria

Participants who experience or have one or more of the following conditions will be excluded:

1. Substance abuse or dependence;

2. Cardiovascular disorder (for example, arrhythmia) or a pacemaker;

3. Neuropsychiatric conditions (for example, epilepsy, depression or panic disorder);

4. Other diabetic microvascular complications (for example, diabetic nephropathy or diabetic retinopathy) within the past three months;

5. HbA1c > 11%;

6. A change in antihyperglycemic medications in the three months prior to screening;

7. A diagnosis of diabetic foot ulcer;

8. The presence of severe pain other than that induced by PDN (for example, arthritis, back pain or headache);

9. Abnormal blood test (HbA1c, blood urea nitrogen, creatinine, thyroid-stimulating hormone, triiodothyronine, free thyroxine, Vitamin B12) or urine test (proteinuria);

10. Neuropathic pain caused by a condition other than DM (for example, malignant disease, tarsal tunnel syndrome, neurothlipsis, vitamin B12 deficiency, hypothyroidism, neurotoxicity (for example, lead, alcohol or smoking), medication (for example, chemotherapy or isoniazid), transient ischemic attack, stroke, multiple sclerosis, chronic inflammatory demyelinating polyneuropathy, uremic neuropathy, sub-acute combined spinal cord degeneration, phantom limb pain or atherosclerosis obliterans);

11. Known hypersensitivity reaction after acupuncture treatment or an inability to cooperate with the acupuncture procedure;

12. Electrical therapy or patch treatment (for example, lidocaine or capsaicin) for PDN used within the past two weeks;

13. Acupuncture, moxibustion, cupping or herbal medicine for PDN used within the past two weeks;

14. Participation in other clinical trials within the past three months;

15. Pregnancy, planning a pregnancy or breast-feeding;

16. Unwillingness to comply with this study protocol.

### Randomization and allocation concealment

Random numbers will be generated using a computerized random number generator through the stratified block randomization method of the SAS package (Version 9.1.3; SAS institute Inc., Cary, NC, USA) with a random block size of three prepared by a statistician (KWK) who is blinded to this trial. A stratified random number will be used according to whether or not a subject is taking pain control medication for PDN. Sequentially numbered, opaque, sealed assignment envelopes will be used to ensure that our allocation is concealed. Each envelope will contain a paper with the name of the group for allocation. Random allocation will be conducted at the second visit for participants who provide informed consent and meet the criteria for inclusion. The two types of treatment, electroacupuncture or sham electroacupuncture, will be explained to the participants as ‘classical electroacupuncture, typically used in Korean medical clinics’ or ‘non-classical electroacupuncture, rarely used in Korean medicine clinics’ [28]. The researcher will open the envelope in front of a participant after determining that the participant meets the criteria for inclusion and that the enrolled participant has completed the baseline assessments. Before identifying the allocation information, the researcher will write the name of the participant and the date on the envelope and the enclosed paper to prevent them from being re-used. The envelopes will be kept in a double-locked cabinet that is managed by a person not involved in the study. To blind other researchers in the study, the allocation information of the two types of acupuncture treatment will be recorded on a practice guide note and be shared only with the acupuncturists.

### Blinding

The participants and the outcome assessors will be blinded to the type of acupuncture, and the data managers, statisticians and study monitors will be blinded to the allocation. The sessions will be organized, if possible, so that the participants from the different groups will not meet. The acupuncturists will not provide any clues about the allocation information to the participants to blind them during the treatment and will not write this information in a patient chart so as to blind the outcome assessors, study monitors and data managers. The researchers will treat the participants without discrimination and will provide information about critical questions according to the pre-scheduled standard operating procedures (SOPs). The blinding will be maintained until the study is completed, at which point all of the data will be collected, all of the queries will be resolved and the database will be locked. For the evaluation of the blinding, the participants will be asked to assess the blinding test after the last treatment.

### Intervention

The electroacupuncture treatment will be conducted by four doctors of Korean medicine (DKMs; SL, JHK, THK and KMS) with more than six years of college education in Korean medicine and at least six years of clinical experience. These doctors are specialists in acupuncture and moxibustion or internists certified by the Korean Ministry of Health and Welfare.

To maintain the same conditions, except for the needling components [29], among the three groups, all of the treatment regimens and procedures will be written in detail as the pre-specified protocol and SOPs. Each practitioner will be allowed to maintain a moderate interaction with the participants: at each visit, the practitioner will ask the participant about their recent symptoms and improvement before starting the treatment and will record the answers in the patient chart; the practitioner will not pursue an excessive physical examination during the pulse-taking or touching the subject's foot and leg and the participant will be allowed to ask about the treatment itself without restriction, but the practitioner will not give confident or positive encouragement to questions about a prognosis. This information will be shared with the practitioners in several workshops to standardize the treatment before the beginning of the study. With the electroacupuncture group and the sham group, two sessions per week will be conducted over eight weeks for a total of 16 sessions. The treatments will be performed with disposable stainless steel needles (0.25 × 40 mm, Dongbang Acupuncture Inc., Chungnam, Republic of Korea) after the acupuncture points are sterilized with a disposable 70% isopropyl alcohol swab. During the treatment, the participant will lie supine with a cushion under the knees to ensure a stable position.

#### Electroacupuncture treatment

A total of 12 acupuncture points at the bilateral ST36, GB39, SP9, SP6, LR3 and GB41 have been selected by the consensus of professors and researchers with an acupuncture and moxibustion specialist license in Korea. The selections are based on a text book [30] and literature reviews [20,31-33], and the selection of a maximum of three individual acupuncture points (among the bilateral ST44, SP2, SP3, BL66, KI1, GB43 or LR2) will be allowed at the discretion of the practitioners. The practitioner will insert the needles in the acupuncture points and induce a deqi sensation. A battery-operated electroacupuncture device (PG-306 pulse generator, Suzuki Iryoki, Tokyo, Japan) will be connected to the handles of each needle, which will remain in place for 30 minutes with a mix of 2 Hz/120 Hz (altering every 2 seconds), at 5 to 10 mA and approximately 80% bearable intensity. The practitioner will regulate the intensity by the degree of the twitching of muscles, or by a request from the subject, and can make one adjustment during the treatment.

#### Sham electroacupuncture treatment

Superficial needling (3 to 5 mm) is performed without a deqi sensation, creating a false movement that is similar to twisting acupuncture at the non-acupuncture points that are matched to each of the real acupuncture points.

1. Medial 1: Point 2 cm below and 2 cm medial from SP9 with 5 mm needling;

2. Medial 2: Point 1.5 cm above and 1.5 cm medial from SP6 with 4 mm needling;

3. Lateral 1: Point 1.5 cm below and 1.5 cm lateral from ST36 with 5 mm needling;

4. Lateral 2: Point 1.5 cm above and 1.5 cm lateral from GB39 with 4 mm needling;

5. Foot 1: Point 1 cm below and 0.2 cm lateral from LR3 with 3 mm needling;

6. Foot 2: Point 1 cm below and 0.2 cm lateral from GB41 with 3 mm needling.

The acupuncture needle is connected to the electroacupuncture device, but no electrical current is passed to the needle which remains in place for 30 minutes. The light is seen and the sound of the pulse generator is heard by the participant as in the electroacupuncture group.

#### Usual care

The usual care group activities include education, counseling and medications for the glucose level and pain control. The participants will receive the educational materials with an explanation of DM and PDN in addition to advice from the DKMs at the second visit. If the participants use oral medication or insulin injection for regulating their glucose levels, then they will be allowed to continue with that medication on the condition of minimal change (≤ ± 25%). If the participants take pain control medications (for example, antiepileptics, SNRIs, tricyclic antidepressants, antidepressants or opioids), then they will be allowed to continue the medication on the condition that no change will be made during the treatment phase. The electroacupuncture and sham groups will also receive the care provided to the usual care group.

#### Co-interventions

Except for the above-stated medications, the participants in all of the groups will not be allowed to use pain control treatments for PDN, such as electrical spinal cord stimulation, transcutaneous electrical nerve stimulation (TENS), laser therapy, patch therapy, herbal medicine, psychotherapy or other acupuncture treatment. The participants will be allowed to take a maximum of 3 g rescue medication (acetaminophen) per day, and this medication will be recorded in a diary.

### Outcome

The detailed outcome measurement time points are provided in Table 1.

**Table 1:** Schedule for treatment and outcome measurements

**Period**	**S**							**T**						**P**					
**Visit**	**1**	**2**	**3**	**4**	**5**	**6**	**7**	**8**	**9**	**10**	**11**	**12**	**13**	**14**	**15**	**16**	**17**	**18**	**19**
**Week**		**1**		**2**		**3**		**4**		**5**		**6**		**7**		**8**		**9**	**17**
Informed consent	●																		
Demographic characteristics	●																		
Inclusion/Exclusion criteria	●																		
Conformity assessment	●																		
Blood test	●																		
Vital signs	●	●	○	○	○	○	○	○	○	●	○	○	○	○	○	○	○	●	●
Medical history	●	●	○	○	○	○	○	○	○	●	○	○	○	○	○	○	○	●	●
Treatment expectancy questionnaire	●																		
Blinding test																	○		
Random allocation		●																	
Treatment		○	○	○	○	○	○	○	○	○	○	○	○	○	○	○	○		
PI-NRS	●	●								●								●	●
SF-MPQ		●								●								●	●
Sleep disturbance score		●								●								●	●
SF-36		●								●								●	●
Beck Depression Inventory		●								●								●	●
PGIC		●								●								●	●
Safety assessment		●	○	○	○	○	○	○	○	●	○	○	○	○	○	○	○	●	●

*P* post-treatment phase, *PGIC* Patient Global Impression of Change, *PI-NRS* 11-point Pain Intensity Numerical Rating Scale, *S* screening phase, *SF-36* Short-Form 36v2 Health Survey, *SF-MPQ* Short-Form McGill Pain Questionnaire, *T* treatment phase, ● All groups, ○ Both the electroacupuncture and sham group.

#### Primary outcome measurement

The primary outcome with respect to the effectiveness for PDN will be the mean change in the PI-NRS (0 = no pain and 10 = worst possible pain, 11-point Likert scale) from baseline to nine weeks. The PI-NRS has been widely used to assess chronic pain intensity in conditions like diabetic neuropathy, postherpetic neuralgia, chronic low back pain, fibromyalgia and osteoarthritis in placebo-controlled clinical trials. Compared with global assessments of change in pain perception, the estimated difference that is of clinical importance is a decrease of approximately two points or approximately 30%, regardless of disease, gender, age, study result or treatment group [34]. From the screening phase, the participants will receive a daily diary to record their average pain-related symptoms on the PI-NRS day (07:00 to 19:00) and night (19:00 to 7:00). The participants will be instructed not to take a rescue medicine before assessing the pain and to bring the diary to the hospital at every visit. If a participant forgets to bring the dairy, then the researchers will call before the next visit to identify whether the participant is recording the diary properly and to request compliance with presenting the diary at each visit. We will assess the participants at the second visit before starting the treatment and at the 5th, 9th and 17th weeks from baseline using the PI-NRS. The mean data during the week prior to these endpoints will be used for the analysis.

#### Secondary outcome measurements

The secondary outcomes include the mean changes in the Short-Form McGill Pain Questionnaire (SF-MPQ), the sleep disturbance score (11-point Likert scale), the Short-Form 36v2 Health Survey (SF-36), the Beck Depression Inventory (BDI) and the Patient Global Impression of Change (PGIC) at the 5th, 9th and 17th weeks from baseline.

The SF-MPQ, developed by R. Melzack in 1987, is a shortened scale of the MPQ. It consists of a 15-descriptors multidimensional questionnaire, the Present Pain Intensity (PPI) index of the standard MPQ and a visual analogue scale (VAS). Fifteen descriptors (11 domains of sensory and four domains of affective descriptors) are ranked on an intensity scale as a four point Likert scale (0 = none, 1 = mild, 2 = moderate or 3 = severe) [35]. The SF-MPQ is more effective in a situation in which the standard MPQ requires excessive time for recording. Various trials for evaluating diabetic neuropathy have used the SF-MPQ to assess the status of pain [36,37]. Kim *et al*. developed a Korean version of the SF-MPQ and assessed its reliability and validity for use with Korean patients [38].

The sleep disturbance score will be recorded in a daily diary on an 11-point Likert scale (0 = no interference with sleep and 10 = complete interference with sleep, unable to sleep because of pain) for evaluating the quality of sleep in the same way that the PI-NRS evaluates pain levels. The symptoms of PDN are worse at night, and patients with PDN have more impairment in their sleep measurements. Sleep impairments are a major cause of the decreased quality of life of patients with PDN. [18]. It is important to measure sleep disturbance to identify the effect of the treatment on the PDN.

The SF-36 will be used for assessing the HRQoL of the PDN at the 5th, 9th and 17th week from the baseline. It is composed of 36 questions in eight domains: physical functioning, role-physical, bodily pain, general health, vitality, social functioning, role-emotional and mental health [39]. It can be completed in 5 to 10 minutes, and higher scores indicate a better HRQoL. The SF-36 is used to measure the functional health and well-being of patients and non-patients. There is a Korean version of the SF-36, and Han *et al*. confirmed its reliability and validity for use in the HRQoL of life measurements with elderly Korean patients [40].

To assess depression, BDI scores will be used at the 5th, 9th and 17th weeks from baseline. The BDI, developed by AT Beck in 1967, is one of the most widely used self-rating scales for measuring the severity of depression. It consists of a 21-question multiple-choice questionnaire to assess depression symptoms of hopelessness and irritability, cognition (for example, guilt and feelings of being punished) and physical symptoms (for example, fatigue and weight loss). Higher total scores indicate a more severe level of depression.

The patient global assessment will be measured by PGIC, a self-reported seven-point categorical scale that is used to evaluate the overall improvement after treatment [34]. Participants subjectively evaluate themselves as to the improvement of their symptoms at 5th, 9th and 17th weeks from baseline by selecting one of the following seven stages: 1) very much improved, 2) much improved, 3) minimally improved, 4) no change, 5) minimally worse, 6) much worse or 7) very much worse.

The Credibility and Expectancy Questionnaire [41] with a nine-point Likert scale will be used to evaluate the treatment expectancy of the participants. At the first visit, the participants will select the scores (1 = not at all, 5 = somewhat and 9 = very much) for the following question, “How much do you feel that the electroacupuncture therapy will help to reduce your symptoms?”

### Sample size

There were no previous clinical trials to evaluate the effect of electroacupuncture for treating PDN with a three-armed RCT. A formal power calculation was not performed because this study is planned to evaluate its feasibility and to calculate a required sample size for subsequent definitive randomized clinical trials. A sample size of 15 per group and a total number of 45 will be included; considering a possible 20% drop out rate, this number of subjects is the recommended minimum number per group to be considered for pilot studies [42].

### Statistical analysis

The study has one primary null hypothesis: there is no difference in the change of the primary endpoint among the three groups after eight weeks of treatment. The intention-to-treat (ITT) analysis as a primary analysis will be performed, including all participants who are randomized and have provided baseline data, and the per-protocol (PP) analysis will be conducted as a reference, including the participants who complete the study following the predefined protocol. The minimum number of treatments for the PP set is more than 12 sessions without three continuous absences from the treatment sessions. The mixed model for repeated measures (MMRM) will be used to handle missing data. The data will be presented as the mean (standard deviations or 95% confidence intervals) or median (quartiles) for continuous data (for example, age and duration of disease) and n (%) for categorical data (for example, gender and whether taking a pain control medication).

For the descriptive analysis, we will perform a two-sample *t*-test or a Wilcoxon rank sum test for continuous data and a chi-squared test or Fisher’s exact test for categorical data, according to whether the data are normally distributed or skewed.

For the confirmatory analysis, an analysis of covariance (ANCOVA) with the baseline measurements as a covariate, the treatment group as a fixed effect, and taking a pain control medication as a stratified variable will be used for the primary outcome (PI-NRS) and secondary outcomes (SF-MPQ, sleep disturbance score, SF-36 and BDI) at the 5th, 9th and 17th weeks from baseline (after randomization). The PGIC and blinding test will be analyzed using a chi-squared test. If a significant difference is identified among the three groups, then multiple comparisons will be conducted to determine which groups are different. ANCOVA without considering stratification will be performed to compare the covariance with the main analysis to determine whether taking a pain control medication is a suitable factor that affects the treatment outcome. The mean change in the values following the treatment in each group will be examined using a paired *t*-test or a Wilcoxon signed rank test, and 95% confidence intervals will be presented. To identify a trend, a repeated measures analysis of variance will be performed for differences over time and among groups. All of the analyses will be performed using a significance level of 5% by SAS statistical software (Version 9.1.3; SAS institute Inc., Cary, NC, USA).

### Data and safety monitoring

Regular monitoring will be conducted to ensure quality control of the data according to the planned protocol and SOPs. Monitors who are blinded to the allocation information will evaluate whether the recruitment procedures are correctly performed and whether the data are adequately recorded according to the protocol in the case report forms. If there are necessary changes in the study methods, such as changes to the eligibility criteria, treatment regimens or duration of follow-up, then the investigators would discuss these issues with independent researchers and statisticians. In the event that severe adverse events and crucial issues occur, the investigators will determine whether these issues are acceptable or if it will be necessary to amend or end the trial.

### Adverse events

Practitioners will record all unexpected and unintended responses that are not necessarily related to electroacupuncture treatment as reported by each participant on an adverse event report form at every visit. The adverse events known to be related to electroacupuncture treatment are pain, dizziness, bleeding, aggravation, dizziness, pain, anxiety and infection [22]. A causal relationship between the electroacupuncture treatment and adverse events will be evaluated using a six-grade scale (1 = definitely related, 2 = probably related, 3 = possibly related, 4 = probably not related, 5 = definitely not related and 6 = unknown), and the seriousness of the adverse events will be scored using a four-point scale (1 = mild, 2 = moderate, 3 = severe and 4 = extremely severe) by the assessment of two researchers (SL, SYJ).

### Participant protections and ethics

The study was planned in accordance with the Helsinki Declaration and the Korean Good Clinical Practice Guidelines to protect the participants and was approved by the institutional review board (IRB) of Daejeon University Hospital (djomc-83). Informing the participants about the potential benefits, risks, alternatives and responsibilities during the study is performed by the researchers through the consent process. To avoid potential adverse events, if the practitioner judges that the participant is not suitable for electroacupuncture treatment due to an abnormal health condition, such as moderate fatigue or a common cold, then the treatment will be rescheduled within three days.

## Discussion

The object of this study is to assess the effectiveness and safety of electroacupuncture for treating PDN. We designed a three-armed parallel study to determine the specific effect of electroacupuncture (the electroacupuncture group versus the sham group) and the overall effect of electroacupuncture (the electroacupuncture group versus the usual care group). Because medical devices tend to have more enhanced non-specific effects than do placebo pills [43,44] and it is difficult to separate some needling components (for example, stimulation of the skin) of acupuncture treatment from sham acupuncture [29], it is necessary to investigate the specific and non-specific effects of acupuncture by including a sham control and the usual care which is applied to the other groups [45].

Conventional usual care includes lifestyle intervention as well as routine antihyperglycemic medications. The Diabetes Prevention Program provides healthy diet and exercise counseling to type 2 DM patients as an intensive lifestyle intervention [46]. One large trial assessing the long-term effects of lifestyle intervention in type 2 diabetes patients emphasized a low-calorie, moderate-fat diet and increased physical activity to reduce cardiovascular disease risk [13]. Another translational trial showed that lifestyle interventions for pre-diabetic neuropathy resulted in partial cutaneous reinnervation and decreased pain [47]. Therefore, education and counseling for diet and exercise are included in our trial as part of usual care.

It is known that electroacupuncture and manual acupuncture operate through different scientific mechanisms [22,48]. A study in rats suggested that manual acupuncture analgesia probably occurred through a contribution from collagen fibers, whereas electroacupuncture analgesia is involved with peripheral nerve receptors [49]. Some studies have suggested that electroacupuncture is more effective than is manual acupuncture in raising the pain threshold and changing the blood flow by causing a greater effect on central endorphins, such as beta-endorphin or dynophins [50-52]. Other studies have reported that manual acupuncture and electroacupuncture have comparable effects [53,54], or that manual acupuncture is superior to electroacupuncture in similar conditions because it more readily elicits deqi sensations, which are conveyed by type III and IV nerve fibers [48].

In diabetic neuropathy, some studies in animal models have shown that electroacupuncture is effective in controlling neuropathic pain and functional deficits [55,56]. One RCT suggested that electroacupuncture is more effective in improving physical activity and QoL and reduces the need for oral analgesic medication than manual acupuncture [31]. Electrical stimulation, such as percutaneous nerve stimulation, was recommended as level B in recent clinical guidelines [3]. Considering these previous examinations and the clinical experience of our DKMs, we decided to use electroacupuncture for this clinical trial.

Frequency is known to be a key electroacupuncture parameter, and the effects of electroacupuncture are different according to the frequency. It is generally recognized that low frequency stimulation (LF) < 10Hz releases opioid peptides, such as beta-endorphin and enkephalin, and has a relatively longer-lasting and generalized effect, while high frequency stimulation (HF) around 80 to 200 Hz releases different opioid peptides, such as dynorphin, and has a relatively short-term and predominantly segmental effect [48]. In practice, it is recommend that both LF and HF be used together as a dense-disperse (DD) wave because the DD mode can prevent nerve accommodation and release various neurotransmitters [22,48]. We planned to apply 2/120 Hz mixed frequencies in the electroacupuncture group.

Most medications for PDN offer only temporary relief from neuropathic pain. However, some etiology-based treatments such as alpha-lipoic acid were reported to improve reduced nerve conduction velocity as well as subjective outcomes [57]. Recently, a randomized sham-controlled trial for PDN showed that acupuncture was more effective than sham acupuncture in both improving nerve conduction velocity and reducing pain [33]. The results indicate that for PDN, acupuncture is superior to placebo control; however, the mechanisms involved in the effects of acupuncture on nerve recovery and microvascular changes in diabetic neuropathy are unknown.

In spite of the large number of studies of acupuncture for PDN, the quality of the reports was limited, and their clinical value cannot be determined [25]. To reduce the risk of bias, we will conduct our study with proper randomization and allocation concealment. In addition, we will use appropriate blinding for the assessors, participants (in the electroacupuncture and sham group), data manager, statisticians and study monitors (of all groups).

This trial will be the first well-designed placebo-controlled RCT to assess the effectiveness and safety of electroacupuncture for treating PDN and to provide the basis for a future large-scale, multicenter trial.

## Trial status

Recruitment for the trial began in June 2012 and will be completed by the end of July 2013. We expect that the results will be reported by the end of 2014.

## Abbreviations

ANCOVA: Analysis of covariance; BDI: Beck depression inventory; CONSORT2010: Consolidated standards for reporting of trials statement 2010; DD: Dense-disperse; DKMs: Doctors of Korean medicine; DM: Diabetes mellitus; DPN: Diabetic peripheral neuropathy; FDA: Food and Drug Administration; HbA1c: Hemoglobin A1c; HF: High frequency stimulation; HRQoL: Health-related quality of life; ITT: Intention-to-treat; IRB: Institutional review board; LF: Low frequency stimulation; MMRM: Mixed model for repeated measures; MNSI: Michigan neuropathy screening instrument; PDN: Painful diabetic neuropathy; PGIC: Patient global impression of change; PI-NRS: 11-point pain intensity numerical rating scale; PP: Per-protocol; PPI: Present pain intensity; RCTs: Randomized clinical trials; SF-36: Short-form 36v2 health survey; SF-MPQ: Short-form McGill pain questionnaire; SOPs: Standard operating procedures; STRICTA2010: Standards for reporting interventions controlled trials of acupuncture 2010; TENS: Transcutaneous electrical nerve stimulation; VAS: Visual analogue scale.

## Competing interests

The authors declare that they have no competing interests.

## Authors’ contributions

SL planned the study protocol and drafted the manuscript. JHK, KMS, JEK and THK made substantial contributions to designing the study protocol. KMS and JEK participated in the critical revision of the manuscript. KWK and ML participated in the design of the statistical analysis. SYJ, MSS, ARK and HJP participated in the design of the outcome measurements and in assessing the outcomes. KEH helped to draft the manuscript. SMC had the final responsibility for the decision to submit for publication. All of the authors have read and approved of the final manuscript.
